# Interactive Network Analytical Tool for Instantaneous Bespoke Interrogation of Food Safety Notifications

**DOI:** 10.1371/journal.pone.0035652

**Published:** 2012-04-18

**Authors:** Tamás Nepusz, Andrea Petróczi, Declan P. Naughton

**Affiliations:** School of Life Sciences, Kingston University, London, United Kingdom; The Australian National University, Australia

## Abstract

**Background:**

The globalization of food supply necessitates continued advances in regulatory control measures to ensure that citizens enjoy safe and adequate nutrition. The aim of this study was to extend previous reports on network analysis relating to food notifications by including an optional filter by type of notification and in cases of contamination, by type of contaminant in the notified foodstuff.

**Methodology/Principal Findings:**

A filter function has been applied to enable processing of selected notifications by contaminant or type of notification to i) capture complexity, ii) analyze trends, and iii) identify patterns of reporting activities between countries. The program rapidly assesses nations' roles as transgressor and/or detector for each category of contaminant and for the key class of border rejection. In the open access demonstration version, the majority of notifications in the Rapid Alert System for Food and Feed were categorized by contaminant type as mycotoxin (50.4%), heavy metals (10.9%) or bacteria (20.3%). Examples are given demonstrating how network analytical approaches complement, and in some cases supersede, descriptive statistics such as frequency counts, which may give limited or potentially misleading information. One key feature is that network analysis takes the relationship between transgressor and detector countries, along with number of reports and impact simultaneously into consideration. Furhermore, the indices that compliment the network maps and reflect each country's transgressor and detector activities allow comparisons to be made between (transgressing vs. detecting) as well as within (e.g. transgressing) activities.

**Conclusions/significance:**

This further development of the network analysis approach to food safety contributes to a better understanding of the complexity of the effort ensuring food is safe for consumption in the European Union. The unique patterns of the interplay between detectors and transgressors, instantly revealed by our approach, could supplement the intelligence gathered by regulatory authorities and inform risk based sampling protocols.

## Introduction

The growing globalization of food supply trade routes satisfying the demand for all year around availability of a wide variety of foodstuff poses a significant challenge for regulations. It necessitates continued advances in control measures to ensure that, as far as possible, citizens enjoy variety with safe and adequate nutrition. The increase in trade routes coupled with advances in information processing methods provides unique opportunities to investigate global food safety by applying new approaches, with the view to aid regulatory officials to enforce expanding food safety regulations. This is exemplified by the recent introduction of an Internet based portal to provide access to the EU based Rapid Alert System for Feed and Food (RASFF) [1]. The new data portal is an advance upon the previous system of issuing weekly alerts in tabular form, published as pdf documents. The new portal allows interactive searches for tailored needs and results are downloadable into Excel spreadsheets. As the database is enormous and rapidly expanding, the portal is arguably most useful where intelligence is available to pinpoint a search. With outputs in the form of tabulated data, any broad search is likely to produce a large data table which will require further processing by, for example, descriptive statistics and graphical representations. Normally some data cleaning and pre-processing (e.g. further categorization) is required to produce useful summaries of the raw data captured in searching the RASFF portal.

Global efforts to secure food safety through information processing can be broadly divided into two categories: i) early identification of emerging incidents and ii) analysis of historical trends by descriptive statistics to inform on potential patterns of concern. Whilst endeavors to spot emerging incidents are at an early stage, many reports have used descriptive statistics approach [2]–[4]. These include a detailed analysis of global food alerts during 2007 for metal contaminants which revealed key countries, metal contaminants, food types and seasonal trends [5]. This approach may be especially useful for an individual country to track a commodity or contaminant but is time consuming and somewhat subjective which undermines comparability. With up to twelve publicly available variables for each notification, and approaching four thousand notifications per year, the power of descriptive statistics is insufficient to interrogate the data for trends without lengthy processing through multiple graphs [2].

To enable in-depth data mining, further reports introduced network analysis to interrogate food notification databases [6]–[8]. The network visualization approach allows facile handling of enormous quantities of data that arise from food notifications in line with the needs of countries that are adopting and implementing food security measures. It provides the ability to instantly access the country-specific components of the several thousand annual reports to enable each country to identify the major transgressors and detectors within its trading network. Using this approach, the major and minor detector countries were readily identifiable along with changing patterns over time [8]. A key feature of the network approach is that the tool provides impact values, recorded as a Detector Index (DI) and Transgressor Index (TI) for each nation. The two versions of DI and TI values are calculated using the two algorithms PageRank [9] and HITS [10]. RASFF notifications were segregated by type into border rejections, information or alerts. Given the varying importance of these notification types, taking these subcategories into consideration and mapping these notification types to the network detector indices could reveal the dominant feature in Detector and/or Transgressor Impact. Thus, it was timely to refine the network tool with the capability to process all notifications or just border rejections for analysis, along with options to focus on a particular type of contamination (e.g. mycotoxins, bacteria or metals).

Therefore the aim of this study was to extend our previous reports on network analysis relating to food notifications [2], [5]–[8] and include an optional filter by type of notification and in case of contaminations, by type of contaminant in the notified foodstuff. This filter allows, for the first time, the simultaneous comparison between types of contaminant for the selected country. These extensions may be used for monitoring purposes where instantaneous interactive maps can be obtained. A new feature is the opportunity to download both TI and DI values in Excel format along with a function that allows users to save the graphs as pictures.

## Methods

The demonstration version of the network tool contains data recorded in the Rapid Alert System for Food and Feed (RASFF) system between May 2003 and August 2008 for all filters but border notifications where data are available up to December 2010. Owing to the fact that the dataset is collated from the EU perspectives, detectors are limited to EU Member States (but they also appear as transgressors if notification was made against their produce) and non-EU countries only appear as transgressors.

The food notification network is created from pairs of countries connected by a food notification logged in the RASFF system. Each pair consists of one country that reports a faulty product from the other country for a given reason at a given time. The network representation depicts these pairwise relationships on the global scale. That is, all pairs active at a selected time point appear on the same graph. Over time, the dynamics of this system are taken into account allowing a report effect to last beyond the date it is logged. This effect decays over time to half of the original effect in 180 days. The calculation method is described in Nepusz et al, 2009 [7].

In order to facilitate comparison between countries, we developed two indices, one for each major role such as detecting and transgressing. These indices are normalized so they can be compared like to like for countries involved. A high transgressor index roughly indicates that a high number of notifications were logged against the country, whereas a high detector index shows a high level of detecting activity evidenced by high number of notifications made. In order to tap into some of the intricacies of the food notification network, we used two different data-mining algorithms: the PageRank [9] and the HITS [10] for calculating two sets of DIs and TIs. The key difference between the two approaches is that the PageRank TIs are independent of the DIs, whereas the HITS indices take each other into account. Therefore in HITS, a DI is high if a high number of notifications is made against a country with high TIs and vice versa. Calculations of these indices are detailed in Nepusz et al. 2009 [7].

Network based indices and numerical expressions of structural properties are described in detail elsewhere [5]–[7]. Briefly, for network plots relating to food safety, the key relationship is the reporting between nations which are termed *edges* and the nations act as *vertices*. Edges have weights reflecting the number of reports between two countries and the elapsed time since the notification date (older notifications having a smaller weight than more recent ones), with detectors appearing as green and transgressors in red. From this point numeric scores can be provided for each contributing nation giving their impact within the database which arises out of number of reports but also the number of countries they link to. Information on the length of paths, degree distributions, and structural properties with number of layers or cluster formations of a given network is capable of revealing important information about the *system* in which actors play upon their individual interest within their opportunities and constrains. A key functionality included the ability to select any time point over the period which initiates an automatic re-graphing of the data given both as trends in impact as transgressors/detectors and as a network map. Because the transgressor and detector indices are normalised, comparison between selected countries is among the available options. The plotted indices, in general, facilitate observing changing trends in each country's activity profile in the context of the entire network.

Filter functions are based on either the type of notification, where border rejections can be separated from all notifications; or major contamination categories identified in the data, namely: metals and two microorganism categories for mycotoxins and bacteria. The categories of contaminants were selected following an assessment of frequency of appearance in the database to reflect the more dominant categories. An additional category for chemicals was created but not built into the current filters. Key components for contamination categories were identified in a subsample based on the number of occurrences. These key components (summarized in Table 1) were used to automatically assign a category to each notification logged. Of the 15,179 notifications, 60.4% were categorized. The remaining 39.6% is comprised of contaminants with low occurrence and notifications owing to incorrect labeling or other non-contamination related infringements upon the EU legislation. The type of notification is categorized as border rejection (as labeled in the RASFF database) and total notifications. The rationale behind allowing users to filter border rejections is that these a major class of notifications and are notifying serious actions which are a mainstay for ensuring food safety within the EU [8]. The uncategorized notifications are included in the ‘all’ function.

**Table 1:** Food notification categories by contamination type and key components used for the filter function (percentage in brackets shows the proportion of the categorized notifications).

Category	Keywords/components
Metal (10.95%)	Arsenic, Lead, Mercury, Cadmium
Microorganism: Mycotoxins (50.45%)	Aflatoxin, Fumonisin, Ochratoxin
Microorganism: Bacteria (20.26%)	Salmonella, Bacillus cereus, Listeria monocytogenes
Chemicals (15.54%)	Benzoic acid, Colour Sudan 1, Methomyl, Nitrofuran, Sulphites
Other (2.80%)	Chloramphenicol, Dioxins, Methamidophos

The additional function of the improved tool is the option to save the visualization map in graphical format or export the indices behind the network map in numerical format, suitable for generating reports. This latter function offers the option to export HITS and PageRank transgressor and detector indices over time. Thus users can select countries of their interest and plot their trend lines for transgressing or detecting activities over time against each other. This is exemplified in a previous report by the authors which was based on data filtered solely for mycotoxin notifications [11].

Colour coding was put in place to assist users. Countries with dominantly detector activities are depicted in green, whereas major transgressors are red. The darkness of the colour corresponds to the strength of that activity (e.g. deep red colour indicates strong transgressing). Countries with a mixed colour (brownish colour resulting from mixing red and green) show activities in both areas. As colouring indicates some strength in either transgressing or detecting, or both, transparently depicted countries can be considered as less-influential actors in the food notification network.

## Results

**Figure 1:**
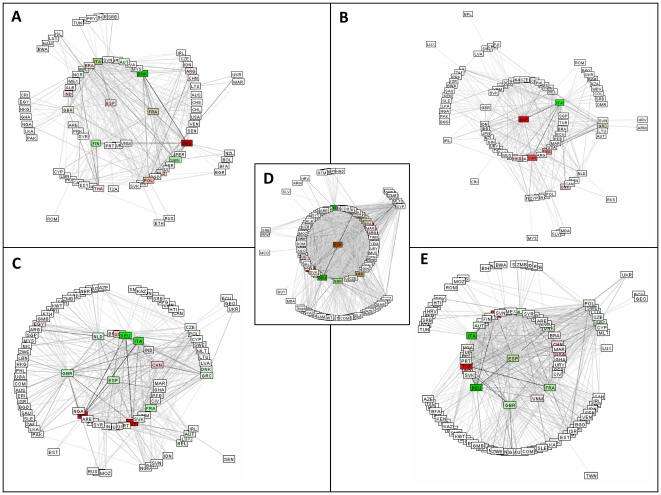
Snapshots of network structures of the RASFF for 2008. Taken on the 1^st^ of January centered on Spain for contaminant categories: (A) Bacteria. (B) Metals. (C) Mycotoxins. (D) All. (E) Microbiological.

A fully functional demonstration version of our analytical tool, operated via a drop down menu for types of contaminant, is on open access via the Internet [http://staffnet.kingston.ac.uk/~ku36087/foodalert/ or to download: http://staffnet.kingston.ac.uk/~ku36087/foodalert/signed-applet_all.html]. More detailed facile analysis of the RASFF database is afforded by application of filters for contaminants by category. Previously we reported that, for the study period, the database may be partitioned by contaminant category with the major classes as follows: heavy metals (10.9%); mycotoxin (50.4%): bacteria (20.3%), with microbiological equaling the sum of mycotoxin and bacteria (70.7%) [11]. **Figure 1** illustrates the network maps for Spain for each of these categories with major detector nations appearing in green and transgressor nations appear in red. For the bacteria category, Spain is highlighted in pink revealing a net transgressor contribution (**Figure 1A**) which become deep red for the metals category (**Figure 1B**) revealing this to be the major concern at the selected time. In contrast, the network map for both mycotoxin and microbiological categories are characterized by a detector profile shown in green.

In addition to the information that can be rapidly generated on the focus nation (Spain) using these maps, a great deal of additional information is readily available. For example, in **Figure 1A**, the major detector nations for notifications relating to bacteria appearing as bright green are ITA, DNK, FIN and SWE. The major transgressor is DEU in bright red with BRA, POL, NLD, ESP, IND and ARG in pink showing they are significant but less serious transgressors for bacteria. FRA appearing as brown signifies a considerable level of both transgressor and detector activities. A key advantage of this approach over other databases is that this rapid deconvolution approach using filters takes seconds and provides the user the results in a processed accessible graphic and tabular form.

**Figure 2:**
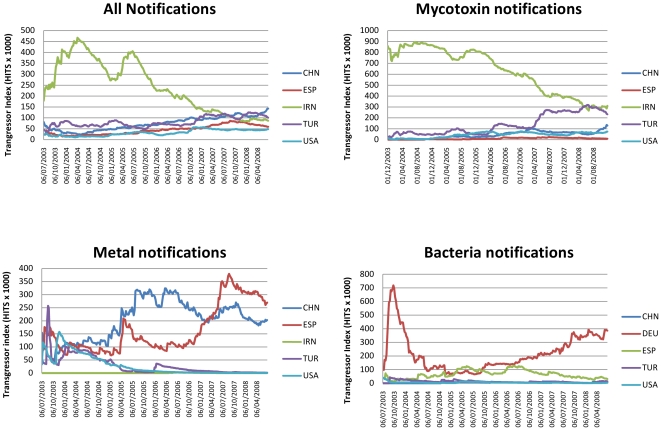
Trend lines in TIs for major transgressors for all RASFF notifications. For clarity the TI indices have been multiplied by 1000.

As the data are processed using network analysis, a download feature is provided to allow users to store and further manipulate selected information. An example of the download feature is given in **Figure 2** which shows the changes in TIs over time for the major transgressor nations for the period studied. The TIs are normalized so the sum of all TI is always equal to 1 at any given time. This approach provides an overview of the contribution of each nation to the RASFF database and may be useful to aid monitoring progress in food safety measures for individual nations. For all notifications IRN was the major transgressor until 2007 which is reflected in the mycotoxin related notifications as they account for some 50% of categorized RASFF notifications [11]. In contrast, for metal related notifications ESP and CHN are the main transgressors with ESP also featuring prominently in the bacteria class.

**Figure 3:**
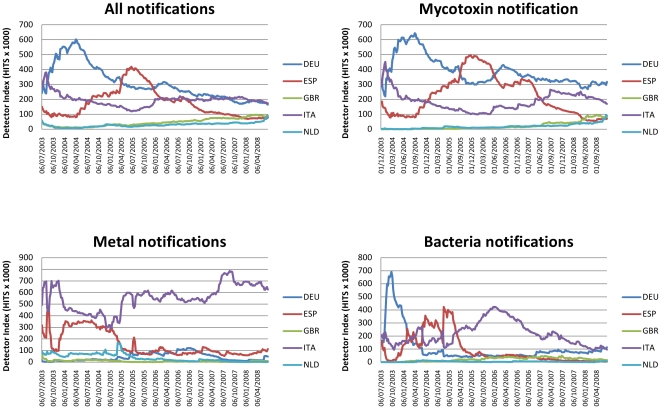
Trend lines in DIs for major detectors for all RASFF notifications. For clarity the DI indices have been multiplied by 1000.

For all notifications, DEU is the predominant detector over the period studied with ITA and ESP making major contributions (**Figure 3**). As expected this pattern holds for mycotoxin notifications as they form some 50% of notifications in our classification. For metal notifications, ITA is the major detector over the entire period with ESP making a strong contribution until mid 2005. Initially NLD and ESP contribute most detector activity for bacteria with ITA predominating for most of the latter years.

The filter approach allows a user to rapidly see the overview and focus in on areas of interest while maintaining control over the wider picture as impact data are normalized and the selected time and nation of focus can be readily and rapidly controlled. As an example, Table 2 gives the TI and DI HITS values on January 2008 showing wide variations in contributions from the top reporting nations in different classes. This is an advance from a previous report which identified the key gatekeeper nations for their overall reports. The current analyses give the breakdowns by category of contaminant for the first time. ITA is a major detection contributor for all categories with relatively minor transgressor activity. In contrast, DEU has largely favourable DIs except for bacteria where it is a strong transgressor with a TI of 344 reflecting a transgressor impact contribution that is ca. one third of all bacteria related notifications at this time. For GBR a good ratio of total DI to TI is less favourable for bacteria and metals. Both NLD and ESP show net transgressor status for metals and bacteria with ESP having almost equality for total TI and DI. Mapping notification types to the network detector indices also revealed that the dominant feature in Detector Impact is the border rejection class (Table 2).

**Table 2:** DI and TI HITS values for major detector nations on January 01, 2008.

	Total	Border Rejection	Metals	Micro-organisms	Mycotoxins	Bacteria
**ITA**						
TI	31.5	0.0	7.6	44.1	28.6	58.9
DI	205.5	224.7	694.3	223.0	228.7	120.4
**DEU**						
TI	35.9	0.5	6.0	44.1	3.6	344.0
DI	175.9	180.8	9.6	262.6	278.6	71.6
**GBR**						
TI	15.9	0.0	5.8	5.4	2.4	16.3
DI	79.9	97.6	0.8	67.2	64.7	26.3
**NLD**						
TI	14.5	0.0	5.8	7.2	0.4	46.4
DI	39.0	44.7	2.9	41.3	41.2	0.5
**ESP**						
TI	78.0	0.0	311.1	32.7	13.1	39.3
DI	71.8	98.7	58.4	66.5	70.3	7.8

*For clarity the DI and TI indices have been multiplied by 1000.

## Discussion

In previous reports, types of contaminants were investigated individually by selecting limited subsets of food safety databases [5], [11]. This report extends significantly on our published network analysis approach to food safety which provides a user-friendly interactive tool to enable regulatory officials and researchers to: i) capture complexity, ii) analyze trends, and iii) identify patterns of reporting activities between countries. Our analysis of RASFF and other food safety databases reveals that intelligence gathering necessitates in depth processing of the extensive dataset well beyond descriptive statistics such as simple frequency counts.

The unique patterns of the interplay between detectors and transgressors, instantly revealed by this approach, could suitably supplement the intelligence gathered by the regulatory authorities and inform risk based sampling protocols. One key feature is that network analysis takes the relationship between transgressor and detector countries, along with volume (number of notifications) and impact simultaneously into consideration. Furthermore, the indices that compliment the network maps and reflect each country's transgressor and detector activities allows comparisons to be made between (e.g. transgressing vs. detecting) as well as within (e.g. transgressing or detecting) activities.

The additional capability of interrogating notification databases as a function of type of notification or by contaminant type affords more focussed analyses by facilitating comparison and contrast between types of contaminants (e.g. metals, mycotoxins, bacteria and total notifications) and notification types (e.g. total versus border rejections). This function revealed a focused role in detection and/or contained problems for individual countries within the global data, which in turn could inform quality control and testing regimes both at the point of origin and at the point of entry to the EU food market.

Even with the limited data (i.e. the publicly accessible information through the RASFF portal or manually collated wordwide reports), we have identified reporting patterns; highlighted important characteristics of the European food safety activities and shown impact of legislations introduced during the observed period. The further capability of selecting the only key border rejection notifications allows rapid scrutiny of arguably the most important sector of the RASFF database.

The usefulness of the network tool evidenced in a clear indication for the remedial measures that were put into place by the EU regulatory authorities (European Commission (EC) 2006) to address the mycotoxin problem with Iran was effective and efficient [12], [13]. Thus, even the limited mycotoxin tool is able to facilitate ongoing monitoring of remedial measures to ensure that they are effective [11]. The further capacity of the novel tool to track a range of contaminants and total versus border rejections considerably enhances this monitoring ability.

In addition, this approach is useful to official bodies to inform their potential forthcoming demands for reference materials as well as for new official controls. For example time demanding collation of data from food safety notifications and presentation by descriptive statistics can be replaced by user friendly network analyses. This approach is currently being explored in the UK [14].

A further key aspect is the inclusiveness of all nations in participation in food safety efforts, including meaningful access and application of notification data. Many nations beyond the boundaries of the EU generate significant amounts of the food consumed in the EU/UK. Attempts to extend participation of these nations in food safety initiatives are paramount. One avenue to enhance uptake and use of global food notifications is to apply state of the art data handling approaches which allow user friendly intelligence gathering along with monitoring capability for current and new safety initiatives.

In summary, this demonstration version of the tool benefits from the addition of filters allows rapid interrogation of RASFF notifications with the instantaneous generation of network maps for the selected time point. The tool provides a level of sophistication in data analysis that is not readily available at present for most stakeholders. The key benefits arise from the ability to select a contaminant category and, via the red and green indicators of a nations' transgressor and detector activity, generate a bespoke report in a matter of minutes using the export download facility. The tool may be used as a ‘stand alone’ instrument or in tandem by informing on detailed scrutiny of complex full databases.

With the vast expansion of global food notifications further steps are warranted in order to apply modern informatics approaches to maintain regulatory efficiency through the datasets. Further work is underway to extend the network tool to: 1) accept periodic food safety feeds and 2) incorporate food safety notifications form global organizations such as the FDA. In the longer term a process control approach is envisaged where a flag system will operate indicating where patterns in key notifications are warrant attention. This ongoing work to incorporate new notifications and additional databases would enable public health agencies and researchers to process food notifications as they are issued from multiple agencies.

Future improvement to the analytical tool will include providing users with structural indices. Clusters, if present in the network, are identified by maximizing a measure called modularity [15]. The optimal partitions corresponding to the maximal modularities were found using the heuristic method [16]. In case the network is organized around a dominant ‘core’ surrounded by nodes that connect to the core only via weak links, we identified countries by their centrality using *k*-core decomposition [17]. To gain insight into the inner structure of these networks, we determined their modularity (whether it can be decomposed into tightly connected smaller subgroups, called clusters). In case of the absence of these networks, we used centrality to determine the role of each country in the network [5]–[7]. Whilst a specific food product (e.g. seafood) network was decomposed into clusters [5], both the worldwide and EU full databases, which include all food reports, exhibited centralised layered structures with key actors in the centre being strongly connected to each other [6], [7].
